# Sequential Clustering Phases for Environmental Noise Level Monitoring on a Mobile Crowd Sourcing/Sensing Platform

**DOI:** 10.3390/s25051601

**Published:** 2025-03-05

**Authors:** Fawaz Alhazemi

**Affiliations:** Department of Computer & Networking Engineering, University of Jeddah, Jeddah 21959, Saudi Arabia; fmalhazemi@uj.edu.sa

**Keywords:** noise pollution, data analysis, noise monitoring, mobile crowd sourcing, mobile crowd sensing

## Abstract

Using mobile crowd sourcing/sensing (MCS) noise monitoring can lead to false sound level reporting. The methods used for recruiting mobile phones in an area of interest vary from selecting full populations to randomly selecting a single phone. Other methods apply a clustering algorithm based on spatial or noise parameters to recruit mobile phones to MCS platforms. However, statistical *t* tests have revealed dissimilarities between these selection methods. In this paper, we assign these dissimilarities to (1) acoustic characteristics and (2) outlier mobile phones affecting the noise level. We propose two clustering phases for noise level monitoring in MCS platforms. The approach starts by applying spatial clustering to form focused clusters and removing spatial outliers. Then, noise level clustering is applied to eliminate noise level outliers. This creates subsets of mobile phones that are used to calculate the noise level. We conducted a real-world experiment with 25 mobile phones and performed a statistical *t* test evaluation of the selection methodologies. The statistical values indicated dissimilarities. Then, we compared our proposed method with the noise level clustering method in terms of properly detecting and eliminating outliers. Our method offers 4% to 12% higher performance than the noise clustering method.

## 1. Introduction

Mobile crowd sourcing/sensing (MCS) platforms are a potential practical approach for reporting noise levels in areas without administered sound pressure level (SPL) meters [1,2,3]. Using MCS platforms for noise monitoring is economically superior to using professional SPL meters. First, MCS does not require prior landscape scanning of an area to identify the appropriate allocation of sound level readers. It depends on the density of mobile phones for area coverage. Second, the MCS does not require installation and operation costs similar to those of the traditional administered SPL meter approach. MCS does not require capital expenses (CapExp) to acquire professional SPL meters or site scanning and engineering to allocate mobile phones (sound meters) [4]. In addition, there are no operation or maintenance (OpExp) costs associated with sound meters (mobile phones) [5,6,7]. Third, mobile phones are better choices than professional SPL meters for monitoring the environment, as they are less expensive (very minor cost overhead) than monitoring systems are. A recent study [8] suggested the use of an MCS platform and mobile phones to monitor bats.

**Figure 1:**
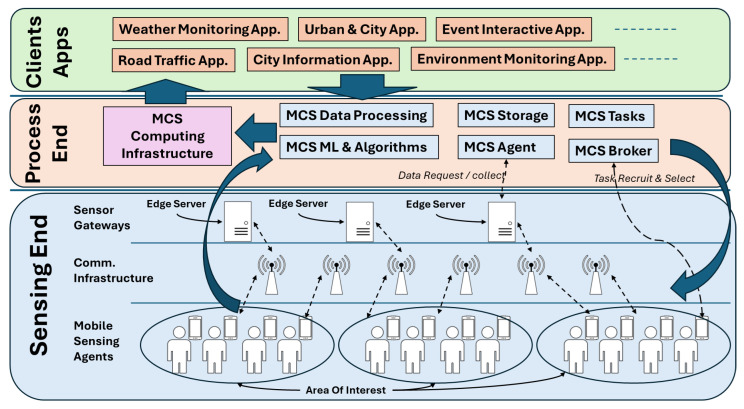
MCS platform architecture.

The MCS approach is an example of a sensor network where the data are gathered through a network of mobile phones, and data processing is applied to translate the readings into noise level observations [9,10,11,12,13,14]. This approach is mainly aligned with technology trends such as the Internet of Things (IoT) and smart cities, which eventually emerged within existing and developed infrastructure [15,16,17,18,19]. The MCS platform architecture is presented in Figure 1 for illustration.

The noise level is an environmental factor that is continuously monitored. Unlike other environmental factors, such as weather conditions, which require few sensors to cover medium- to large-sized areas, monitoring noise levels requires many sensors to cover comparable areas. The reason behind this is the relationship between the noise level and the distance from the noise source. Most MCS platforms that are used in noise monitoring involve gathering samples and readings from large sets of mobile phones, and several data analyses are used to calculate the noise level. Despite the many methodologies in the literature (as discussed in the following Section 2) that select the full population or subset of mobile phones in the area to be engaged in the noise level calculation, we argue that the noise level is not like other environmental factors in which the variations in mobile phone readings are small or neglected. A mobile phone’s location and distance from a sound source (or noise source) have a large influence on mobile readings. If these mobile phones are scattered across an area and faced with a single source of noise, they will report different noise levels. In the literature, some works have suggested the use of spatial clustering, and others have suggested the use of noise level clustering. These solutions have the advantages of capturing the noise level with higher accuracy while completely removing outlier mobile phones from the calculations.

In this work, we hypothesize and explore the use of an MCS platform as a noise-monitoring system and the risk associated with the reporting noise level via impetuous statistical analysis. We propose two sequential clustering phases for noise level monitoring on the MCS platform. Our proposed method can eliminate outlier mobile phones from being considered in the calculation of noise level. In addition, we conducted a real-world experiment with 25 mobile phones in an indoor environment to monitor the noise level. We focused on our experiment to evaluate two major aspects. First, we validated that considering a full population of mobile phones is not an ideal method for reporting noise levels; however, cluster-based methods, including our proposed method, show large variations compared with full populations. According to the *t* test used in the evaluation of the variation, the reported values show that large variations between the full population and other methods are very clear and necessitate clustering mobile phones and eliminating outliers prior to calculating the noise level. Second, we compared our proposed method with two other cluster-based methods in terms of how many outliers have been detected and eliminated from the calculation. Our method offers 4% to 12% higher performance than other methods in terms of detecting and eliminating outliers from the calculation. Our proposed method was designed to detect and eliminate more than 20% of mobile phones from reporting outlier readings.

The structure of this paper is as follows. The background and related works are presented in the Section 2. The problem formulation is outlined in the Section 3. The proposed sequential phases for noise level clustering are discussed in the Section 4, and the experiment is presented in the Section 5. In the Section 6 and Section 7, we report the results, followed by a discussion. Finally, we conclude with our remarks in the Section 8.

## 2. Background and Related Works

### 2.1. System Architecture

The system architecture of the MCS platform is shown in Figure 1. Fundamentally, the basic layering components of the MCS platform are the *sensing end*, *Process End*, and *Client Applications*.

The *sensing end* layer (the bottom layer) consists of three main entities. First, mobile sensing agents, i.e., participant users with mobile phones, sense the environment and collect data. Next, sensor gateways or edge servers receive the sensed data from mobile phones and send the data to the MCS platform’s processing end (upper layer). The communication infrastructure between mobile phones and sensor gateways could include wireless networks, cellular networks, Bluetooth, or any mobile communication media.

The *Process End* layer (the middle layer) is the core of the MCS platform and consists of several components. In this paper, we note the fundamental components. This layer includes *MCS Tasks*, an *MCS Agent*, *MCS Broker*, *MCS Storage*, *MCS Data Processing*, *MCS ML and Algorithm*, and *MCS Computing Infrastructure*. Each MCS platform has a task manager (*MCS Tasks*), which receives requests from Client Applications (upper layer) and schedules them for performing. On the MCS platform, the received tasks initially include the area of interest where environmental or nonenvironmental parameters need to be monitored or reported. The *MCS Broker* receives the tasks along with the specified area of interest and starts to recruit and select participants, i.e., mobile phones, to perform crowd sensing. Once the recruiting and selection is completed, the *MCS Agent* begins to request and collect data from these mobile phones. The collected data are stored in the *MCS Storage*, and further data processing may be applied via *MCS Data Processing* for data representations and transformations. Many MCS platforms adopt machine learning and optimization algorithms to improve data acquisition from crowds. Therefore, a common component is always present in this *Process End* layer, and we concisely name it the *MCS ML and Algorithms*. The main function of this component is to improve and adapt the quality of the reported data on the MCS platform. Finally, the computing facility or the back-end computing power is presented as the *MCS Computing Infrastructure*. Practically, MCS platforms use cloud-based computing servers as a computing infrastructure for easy access and powerful computing facilities.

The last and upper layer is the *Client Application* layer, which holds the application of the MCS platform as a sensing platform. Examples of applications include weather monitoring, road traffic, urban and city applications, and interactive event applications.

A noise-monitoring system is a Client Application that uses an MCS platform as a sensing platform. Previous works have suggested that the *MCS Broker* selects participant mobile phones via different methods, as discussed in Section 3.2.

### 2.2. Trends and Existing Works

MCS is a trending platform for sensing and monitoring systems to support smart cities and the Internet of Things (IoT). An MCS platform consists of and depends primarily on mobile and smart devices to sense and collect measured data from the surroundings and send the data to a collector or storage for further processing. The MCS platform serves as infrastructure to support management information systems (MISs) for monitoring and surveilling smart cities and the environment [20,21,22,23,24], as shown in Figure 1. A multitask framework has been suggested to support multiple types of data collection for different applications, such as environmental monitoring [24,25,26], transportation [27,28,29,30], health services [31,32,33], and public safety [34,35,36,37].

One of the trending and major applications that uses the MCS platform is monitoring noise pollution (noise level). The literature is rich in proposals that use mobile MCS platforms to sense sound levels with different techniques and goals [10,11,12,13,14,38,39,40,41,42]. Noise pollution has been monitored at the university campus level via “NoiseCapture” [11] and at the city level [13,42]. The gathered data were analyzed via spatial analysis [10,41], and further clustering algorithms were used for data analysis [14]. A noise calibration technique was used to increase mobile phone participation in an MCS platform for noise monitoring [40]. An edge-based computing system was explored to support an MCS platform in noise monitoring [12]. An open MCS platform architecture [38,43,44,45] was suggested to support noise monitoring.

### 2.3. Factors Affecting MCS Platforms

While existing MCS-based noise-monitoring systems offer valuable insights, they often suffer from inaccuracies in data statistics reporting. Relying on unfiltered or unanalyzed collective data can lead to erroneous conclusions. Several factors contribute to these issues:

- **Mobile density:** The number of participating devices significantly influences the reported noise levels.
- **Centroid locations:** The geographical centers of mobile phones can skew readings, particularly in unevenly populated areas.
- **Population distributions:** Full populations and clustered groups may yield different noise measurements.
- **Outlier influences:** Individual devices with extreme noise readings can disproportionately affect overall reports.

In Table 1, we review several major studies on MCS-based noise-monitoring systems. In this review, we consider the variations in the abovementioned factors that contribute to the inaccuracy of MCS-based platforms in noise-monitoring systems.

## 3. Problem Formulation

The studies listed in Table 1 suggest that there is an important need for further study of MCS platforms for noise-monitoring systems, particularly considering the statistical methodology. Therefore, in this case study, we explore the influence of mobile phone diffusion in an indoor environment on the accuracy and performance of an MCS platform for noise level reading. We used a sound source with two frequencies to generate two SPLs. To ensure reliable recommendations, this study prioritizes an accurate interpretation of the noise level on the basis of the MCS platform for reading the noise level.

### 3.1. Acoustic Properties

On the basis of the acoustic properties, the distance and angular position of a participant’s mobile phone would have a greater impact on the sound (noise) level readings, and if all the mobile phone readings were collectively calculated, the impact would be worse. Regardless of the number of participant mobile phones, we expect that the distance and angular position of the participant mobile phones would be noticeable in noise level calculations. As a result, outliers are formed, and abnormal readings (from these outliers) are observed in the reported readings. Referring to Table 1, the last column shows that most works included outliers in the readings, which indicates that existing works are misled by the incorporation of outliers in noise monitoring.

To clearly understand why some mobile phones are considered outliers when we use them for noise reading on the MCS platform, we note the nature of the sound level. The sound level is formed as follows:

- Sound pressure (*P*) at the receiver.
- Sound power (*W*), which is the amount of energy emitted by the source of the sound.
- Sound intensity (*I*), which is the rate of sound energy transfer per unit area in the direction of propagation.

According to [53], the relationship can be mathematically represented by Equation (1):

(1) $$I=\frac{W}{A},$$

where *A* is the area (in m^2^), *W* is the power in Watts, and *I* is Watts/m^2^. Furthermore, the sound pressure level (in Pascal) varies according to the distance from the sound source. According to [53], the sound intensity level ($L_{I}$) is found via Equation (2) below:

(2) $$L_{I}=10log_{10}\left(\frac{I}{I_{0}}\right),$$

where *I* and $I_{0}$ are the sound intensity at the recipient point and the sound intensity reference value ($I_{0}=10^{-12}$ Watts/m^2^), respectively. According to [54], the level of sound pressure ($L_{P}$) and the level of sound intensity are equal, as shown in the derived Equation (3)

(3) $$L_{P}=10log_{10}{\left(\frac{P}{P_{0}}\right)}^{2}=10log_{10}{\left(\frac{I}{I_{0}}\right)}^{2}=L_{I},$$

where *P* and $P_{0}$ are the sound pressure level at the recipient point and the reference value for the sound pressure level ($P_{0}=2\times 10^{-5}$ N/m^2^), respectively. The sound pressure level (or SPL) is the level measured by sound meters (called SPL meters), and it is measured instantaneously and reported. It is important to distinguish between the major time-varying noise levels that can be reported, namely, continuous noise, intermittent noise, and impulsive noise. Continuous noise is a constant noise over time; intermittent noise is a noise that appears (heard) and disappears (not heard) irregularly; and impulsive noise is sharp noise such as breaking glass or a gunshot. Among these three levels, continuous noise is the common level that covers MCS platforms, and in research, the other two levels can be considered in the integral of Equation (4) as follows. According to [53], the level of continuous equivalent noise, which is reported over a period (*T*), is shown in Equation (4) below.

(4) $$L_{eq,T}=10log_{10}\frac{1}{T}\int_{0}^{T}\left(\frac{P(t)}{P_{0}}\right)dt,$$

where P(t) is the instantaneous pressure level of the sound reported by the SPL meter (on the MCS platform, it is reported by a mobile phone).

Many existing MCS platforms used in smart buildings and smart cities (including urban areas) have been shown to monitor environmental parameters; however, they lack accurate reports of noise levels. The lack of understanding of the impact of sound parameters such as intensity, power, and pressure within these MCS platforms has led to inaccurate or incomplete noise readings. From Equations (1) and (2), we can infer that the space and distance (*A*) influence the sound intensity level ($L_{I}$), which is reflected and observed by measuring the sound pressure level ($L_{P}$), as in Equation (3), when SPL meters (or mobile phones in MCS platforms) are used. Consequently, when the MCS platform monitors the noise level over a period *T*, i.e., the continuous equivalent noise level ($L_{eq,T}$), the reported noise level has accumulated the abovementioned influences. On the basis of the position of the mobile phone and how it receives the energy of the noise (sound pressure level), the noise level varies drastically between mobile phones even when they are located within a small area (see the Results Section 6).

### 3.2. Existing MCS Methodologies

Assume that we have a set of mobile phones $M=\{m_{1},\;\ldots ,m_{n},\;\ldots ,m_{m}\}$ in the area of interest. Noise monitoring via the MCS platform generally involves monitoring noise from all mobile phones (*M*) in the area of interest; however, existing works suggest that not all mobile phones are recruited for data gathering. The *MCS Broker* is adjusted to recruit a certain number of mobile phones in the area of interest via the following methods.

#### 3.2.1. Full Population (FP)

The full population method (FP) calculates the average reading among all mobile phones in the area of interest. Regardless of the size of the area of interest, all mobile phones are recruited by the *MCS Broker*, and they are considered in calculating the noise via *MCS Data Processing* [55]. The noise level is calculated via the following Equation (5):

(5) $$L_{eq,T}=\frac{\sum_{i=1}^{n}L_{eq,T}^{i}}{n},\;m_{n}\in M$$

where *n* is the number of mobile phones ($m_{n}$) among all mobile phones (*M*) in the area of interest, and the noise level of $m_{n}$ mobile phones is $\left(L_{eq,T}^{n}\right)$. The number of mobile phones *n* must be a positive integer number in Z to avoid dividing by zero, that is, at least one mobile phone must be recruited and report the noise level.

#### 3.2.2. Randomly Selected Single Mobile (RS)

The randomly selected single-mobile phone method (denoted RS) involves randomly selecting and recruiting a mobile phone among all mobile phones in the area of interest. This mobile phone will report the noise level on the basis of its reading (or acquisition from a nearby mobile phone) for the noise level, and this reporting is for all mobile phones in the area of interest [9]. This random selection can be reevaluated by the *MCS ML and Algorithm*, and another random selection by the *MCS Broker* occurs. The noise level in this method is calculated via the following Equation (6), which is essentially Equation (4) for the chosen mobile phone.

(6) $$L_{eq,T}=L_{eq,T}^{r},\;m_{r}\in M$$

where *r* is the index of the randomly selected mobile phone among mobile phones (*M*) in the area of interest.

#### 3.2.3. Subset Selection (SS)

The subset selection method (denoted as SS) selects the set $S=\{m_{p},\;\ldots ,m_{s},\;\ldots ,m_{q}\}$ of mobile phones out of the mobile phones (*M*) in the area of interest, that is, (S⊂M). This selection phase is conducted by the *MCS ML and Algorithm*, which evaluates the population and identifies the subset to be recruited by the *MCS Broker*. Afterward, *MCS Data Processing* calculates the average among the recruited set *S* of mobile phones and considers it to be the reading of the noise level for all mobile phones in the area of interest [39,56,57]. The *MCS ML and Algorithm* continues the evaluation of the number (or size) of the selected subset, and it always keeps adjusting the number to the minimum required number. The metric used to confirm the size of the subset is the quality of standard division, as explained in [57]. The noise level in this method is calculated via the following Equation (7).

(7) $$L_{eq,T}=\frac{\sum_{i=1}^{s}L_{eq,T}^{i}}{s},\;m_{s}\in S,\;S\subset M$$

where *s* is the number of mobile phones ($m_{s}$) in the subset (*S*) selected from the mobile phones (*M*) in the area of interest.

#### 3.2.4. Spatial Clustering (C_spatial_)

The spatial clustering method (denoted as C_spatial_) applies an unsupervised clustering algorithm (in this work, we consider DBSCAN as a clustering algorithm) via the *MCS ML and Algorithm* on the full population of mobile phones in the area of interest. Next, the *MCS ML and Algorithm* will form multiple focused subsets $C=\{c_{1},\;\ldots ,c_{X},\;\ldots ,c_{z}\}$ based on the spatial distance, which are used as subpopulations to be reported to the *MCS Broker* for recruiting, as in Equations (8) and (9) below. In addition, the *MCS ML and Algorithm* forms a set of outliers $P_{spatial}=\left\{p_{1},\;\ldots ,p_{j}\right\}$, which are discarded from the recruitment process by the *MCS Broker*.

(8) $$C=\{c_{1},\;\ldots ,c_{X}\ldots ,c_{z}\},\;z\in Z$$

(9) $$c_{X}=\left\{m_{U},\;\ldots ,m_{X},\;\ldots ,m_{V}\right\},\;m_{X}\in M,\;c_{X}\subset M$$

where *z* is the number of formed clusters in the area of interest, $c_{X}$ is the number of formed clusters, and $m_{X}$ is the number of mobile phones in the formed cluster $c_{X}$ among the formed clusters *C* in the area of interest.

Afterward, the *MCS Data Processing* calculates the average among the recruited mobile phones in the formed clusters $c_{X}$ and considers it the reading of the noise level for all mobile phones in the formed cluster $c_{X}$ in the area of interest. Therefore, we have multiple noise level readings ($L_{eq,T}^{C}$) for the area of interest, as expressed in the following Equation (10):

(10) $$L_{eq,T}^{C}=\left[\begin{array}{c} L_{eq,T}^{c_{1}}\\ \vdots \\ L_{eq,T}^{c_{X}}\\ \vdots \\ L_{eq,T}^{c_{z}} \end{array}\right]=\left[\begin{array}{c} \frac{\sum_{i=U}^{V}L_{eq,T}^{i}}{SizeOf(c_{1})},\;m_{i}\in c_{1},\;c_{1}\subset M\\ \vdots \\ \frac{\sum_{i=U}^{V}L_{eq,T}^{i}}{SizeOf(c_{X})},\;m_{i}\in c_{X},\;c_{X}\subset M\\ \vdots \\ \frac{\sum_{i=U}^{V}L_{eq,T}^{i}}{SizeOf(c_{z})},\;m_{i}\in c_{z},\;c_{z}\subset M \end{array}\right]$$

where $SizeOf(c_{X})$ is the number of mobile phones in cluster $C_{X}$, *i* is the number of mobile phones ($m_{i}$) among mobile phones in the formed cluster ($c_{X}$) for the mobile phones (*M*) in the area of interest, and the set of noise levels ($L_{eq,T}^{C}$) holds the reported noise levels per formed cluster ($c_{X}$).

#### 3.2.5. Noise Level Clustering (C_Noise_)

The noise level clustering method (denoted as C_Noise_) applies an unsupervised clustering algorithm (again, we use DBSCAN as a clustering algorithm) via the *MCS ML and Algorithm* on the full population of mobile phones in the area of interest. Next, the *MCS ML and Algorithm* forms multiple focused subsets $L=\{l_{1},\;\ldots ,l_{Y},\;\ldots ,l_{w}\}$ on the basis of the noise level variation, which are used as subpopulations to be reported to the *MCS Broker* for recruiting, as expressed in Equations (11) and (12). In addition, the *MCS ML and Algorithm* forms a set of outliers $Q_{noise}=\left\{q_{1},\;\ldots ,q_{k}\right\}$, which are discarded from the recruitment process by the *MCS Broker*.

(11) $$L=\{l_{1},\;\ldots ,l_{Y}\ldots ,l_{w}\},\;w\in Z$$

(12) $$l_{Y}=\left\{m_{g},\;\ldots ,m_{F},\;\ldots ,m_{h}\right\},\;m_{F}\in M,\;l_{Y}\subset M$$

where *w* is the number of formed clusters in the area of interest, $l_{Y}$ is the number of formed clusters, and $m_{F}$ is the number of mobile phones in the formed cluster $l_{F}$ among the formed clusters *L* in the area of interest.

Afterward, the *MCS Data Processing* calculates the average among the recruited mobile phones in the formed clusters (all the mobile phones except the outlier mobile phones) and considers it as the reading of the noise level for all the mobile phones in the area of interest. Therefore, we removed the outliers from the noise reading. The noise level is given in Equation (13) below.

(13) $$L_{eq,T}^{L}=\frac{\sum_{Y=1}^{w}\sum_{F=g}^{h}L_{eq,T}^{F}}{\sum_{Y=1}^{w}SizeOf(l_{Y})},\;w\in Z,\;m_{F}\in l_{Y},\;l_{Y}\subset M$$

where $SizeOf(l_{Y})$ is the number of mobile phones in cluster $l_{Y}$, *F* is the number of mobile phones ($m_{F}$) among mobile phones in the formed cluster ($l_{Y}$) in the mobile phones (*M*) in the area of interest, and the noise level ($L_{eq,T}^{L}$) holds the reported noise level for the area of interest without including the outlier mobile phones $Q_{noise}$.

## 4. Sequential Clustering Phases for Noise Level Monitoring on the MCS Platform

In this paper, we propose applying sequential clustering phases to the area of interest prior to calculating the noise level on the MCS platform. We apply the clustering algorithm to the area of interest in repeated phases, namely, the spatial clustering phase and the noise level clustering phase. The proposed method (denoted as C_Spatial_⇒C_Noise_) is implemented at the *MCS ML and Algorithm*, and the two phases of the clustering are applied in sequence.

### 4.1. System Model

The system model for the proposed sequential phases for noise level clustering is as follows. In the first phase, we apply the DBSCAN cluster algorithm for spatial clustering to form multiple clusters ($C_{spatial}=\left\{c_{spatial,1},\;\ldots ,c_{spatial,X},\;\ldots ,c_{spatial,z}\right\}$). In this phase, the proposed approach is formed, in addition to the clusters ($C_{spatial}$) and the outlier set ($P_{spatial}=\left\{p_{1},\;\ldots ,p_{j}\right\}$), which are discarded from the recruitment process by the *MCS Broker*. As a result of this spatial clustering phase, the *MCS ML and Algorithm* forms a list of clusters and their set of mobile phones. From Equations (8) and (9), the formed clusters are expressed in Equations (14) and (15):

(14) $$C^{spatial}=\left\{c_{1}^{spatial},\;\ldots ,c_{X}^{spatial},\;\ldots ,c_{z}^{spatial}\right\},\;z\in Z,\;c_{X}^{spatial}\subset M$$

(15) $$c_{X}^{spatial}=\left\{m_{U},\;\ldots ,m_{X},\;\ldots ,m_{V}\right\},\;m_{X}\in M,\;c_{X}^{spatial}\subset M.$$

The outliers eliminated in this spatial clustering phase are expressed in Equation (16):

(16) $$P^{spatial}=\left\{p_{1}^{spatial},\;\ldots ,p_{j}^{spatial}\right\}$$

In the second phase, we apply noise level clustering to each cluster to eliminate any outlier mobile phones ($Q_{noise}=\left\{q_{1},\;\ldots ,q_{k}\right\}$) from the formed clusters ($C^{spatial}$). The DBSCAN algorithm is reapplied to each cluster ($C_{X}^{spatial}$) to form tuned clusters, as expressed in Equation (17) below.

(17) $$C^{spatial,noise}=\left\{c_{1}^{spatial,noise},\;\ldots ,c_{X}^{spatial,noise},\;\ldots ,c_{z}^{spatial,noise}\right\},\;z\in Z,\;c_{X}^{spatial,noise}\subset M$$

The set of mobile phones to be recruited by the *MCS Broker* is expressed in Equation (18):

(18) $$c_{X,Y}^{spatial,noise}=\left[\begin{array}{c} m_{1,1},\;\ldots ,m_{1,Y},\;\ldots ,m_{1,w}\\ \vdots \;\;\vdots \;\;\vdots \\ m_{X,1},\;\ldots ,m_{X,Y},\;\ldots ,m_{X,w}\\ \vdots \;\;\vdots \;\;\vdots \\ m_{z,1},\;\ldots ,m_{z,Y},\;\ldots ,m_{z,w} \end{array}\right]\subset M$$

In addition, the outliers eliminated in this noise level clustering phase are expressed in Equation (19).

(19) $$Q^{noise}=\left[\begin{array}{c} Q_{1}^{noise}\\ \vdots \\ Q_{X}^{noise}\\ \vdots \\ Q_{z}^{noise} \end{array}\right]=\left[\begin{array}{c} \left\{q_{1,1}^{noise},\;\ldots ,q_{1,Y}^{noise},\;\ldots ,q_{1,w}^{noise}\right\}\\ \vdots \\ \left\{q_{X,1}^{noise},\;\ldots ,q_{X,Y}^{noise},\;\ldots ,q_{X,w}^{noise}\right\}\\ \vdots \\ \left\{q_{z,1}^{noise},\;\ldots ,q_{z,Y}^{noise},\;\ldots ,q_{z,w}^{noise}\right\} \end{array}\right]\subset M$$

Finally, the *MCS Data Processing* calculates the noise levels ($L_{eq,T}^{spatial,noise}$) for the generated and filtered clusters ($C^{spatial,noise}$) via Equation (20).

(20) $$L_{eq,T}^{spatial,noise}=\left[\begin{array}{c} L_{eq,T,1}^{spatial,noise}\\ \vdots \\ L_{eq,T,X}^{spatial,noise}\\ \vdots \\ L_{eq,T,z}^{spatial,noise} \end{array}\right]=\left[\begin{array}{c} \frac{\sum_{i=U}^{V}L_{eq,T,1,i}}{SizeOf(c_{1}^{spatial,noise})},\;m_{i}\in c_{1}^{spatial,noise},\;c_{1}^{spatial,noise}\subset M\\ \vdots \\ \frac{\sum_{i=U}^{V}L_{eq,T,X,i}}{SizeOf(c_{X}^{spatial,noise})},\;m_{i}\in c_{X}^{spatial,noise},\;c_{X}^{spatial,noise}\subset M\\ \vdots \\ \frac{\sum_{i=U}^{V}L_{eq,T,z,i}}{SizeOf(c_{z}^{spatial,noise})},\;m_{i}\in c_{z}^{spatial,noise},\;c_{z}^{spatial,noise}\subset M \end{array}\right].$$

The set of outliers that are not included in the calculation of the noise level via *MCS Data Processing* is a combination of the outliers in Equations (16) and (19) and is generally expressed in Equation (21).

(21) $$Outliers=\left\{P^{spatial},Q^{noise}\right\}=\left[\begin{array}{c} P^{spatial}\\ Q_{1}^{noise}\\ \vdots \\ Q_{X}^{noise}\\ \vdots \\ Q_{z}^{noise} \end{array}\right]=\left[\begin{array}{c} \{p_{1}^{spatial},\;\ldots ,p_{j}^{spatial}\},\\ \{q_{1,1}^{noise},\;\ldots ,q_{1,Y}^{noise},\;\ldots ,q_{1,w}^{noise}\},\\ \vdots \\ \{q_{X,1}^{noise},\;\ldots ,q_{X,Y}^{noise},\;\ldots ,q_{X,w}^{noise}\},\\ \vdots \\ \left\{q_{z,1}^{noise},\;\ldots ,q_{z,Y}^{noise},\;\ldots ,q_{z,w}^{noise}\right\} \end{array}\right]$$

In this model, as presented in Equation (20), the noise level is reported as multiple noise levels, and the mobile phones that are not selected (recruited) by the *MCS Broker* are given in Equation (21). The extreme cases in this model are as follows.

#### 4.1.1. All Counted Cases

In this case, after *the MCS ML and Algorithm* performs the two clustering phases, the set of recruited mobile phones is essentially all possible mobile phones in the area of interest. In the system model, after the execution of the two clustering phases, we have the following. The set of mobile phones to be recruited by the *MCS Broker* is expressed in Equation (22):

(22) $$c_{X,Y}^{spatial,noise}=\left[\begin{array}{c} m_{1,1},\;\ldots ,m_{1,Y},\;\ldots ,m_{1,w}\\ \vdots \;\;\vdots \;\;\vdots \\ m_{X,1},\;\ldots ,m_{X,Y},\;\ldots ,m_{X,w}\\ \vdots \;\;\vdots \;\;\vdots \\ m_{z,1},\;\ldots ,m_{z,Y},\;\ldots ,m_{z,w} \end{array}\right]=M$$

and the set of outliers, which is an empty set; this noise level clustering phase is expressed in Equation (23).

(23) $$Outliers=\{P^{spatial},Q^{noise}\}=\phi$$

#### 4.1.2. All Outliers

In this case, after *the MCS ML and Algorithm* performs the two clustering phases, the set of recruited mobile phones is empty, and all the mobile phones are considered outliers. In the system model, after the execution of the two clustering phases, we have the following. The set of mobile phones, which is an empty set, to be recruited by the *MCS Broker* is expressed in Equation (24):

(24) $$c_{X,Y}^{spatial,noise}=\phi$$

and the set of outliers eliminated in this noise level clustering phase is expressed in Equation (25):

(25) $$Outliers=\left\{P^{spatial},Q^{noise}\right\}=\left[\begin{array}{c} P^{spatial}\\ Q_{1}^{noise}\\ \vdots \\ Q_{X}^{noise}\\ \vdots \\ Q_{z}^{noise} \end{array}\right]=\left[\begin{array}{c} \{p_{1}^{spatial},\;\ldots ,p_{j}^{spatial}\},\\ \{q_{1,1}^{noise},\;\ldots ,q_{1,Y}^{noise},\;\ldots ,q_{1,w}^{noise}\},\\ \vdots \\ \{q_{X,1}^{noise},\;\ldots ,q_{X,Y}^{noise},\;\ldots ,q_{X,w}^{noise}\},\\ \vdots \\ \left\{q_{z,1}^{noise},\;\ldots ,q_{z,Y}^{noise},\;\ldots ,q_{z,w}^{noise}\right\} \end{array}\right]=M$$

### 4.2. Workflow Process

**Figure 2:**
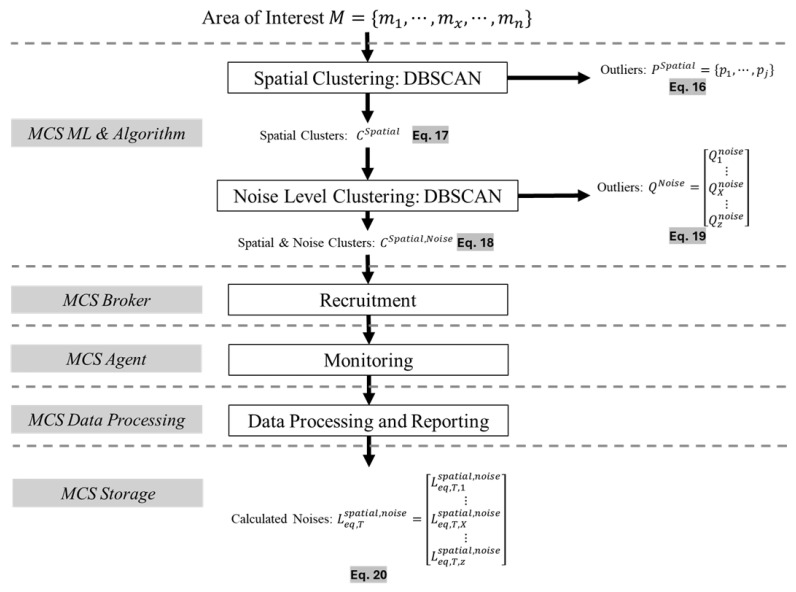
The proposed workflow process.

The workflow process of the proposed approach shown in Figure 2, and it is executed at the *MCS Process End* layer in the MCS platform system architecture. The workflow process is initiated after the application (in our work, the Client Application is the noise-monitoring system) identifies the area of interest to be under observation and monitoring. The *MCS ML and Algorithm* performs two clustering phases, namely, spatial clustering and noise level clustering. Then, it generates the appropriate focused clusters and outliers, as expressed in Equations (16)–(19). Next, the *MCS Broker* recruits the selected mobile phones with respect to their relevant cluster set. The *MCS Agent* then starts the monitoring and reporting task continuously. Afterward, *MCS Data Processing* calculates the noise levels via the given Equation (20) for the observed area of interest and records them at the data repository via *MCS Storage*.

## 5. Experiment

The experimental design included a sound source and 25 mobile phones placed in a room. The experiment runs for 1 min and 40 s (100 s), and all noise readings are available online at [58]. The details of the experiment are as follows.

### 5.1. Sound Source

**Figure 3:**
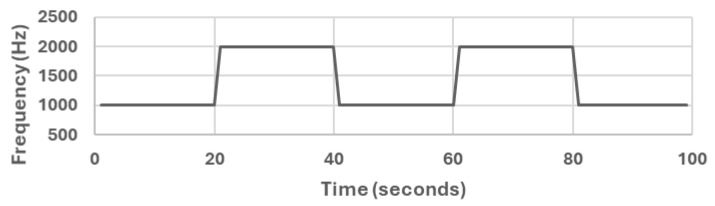
The sound is generated via a sine wave for two frequencies: 1 kHz and 2 kHz.

We used a generated sound audio file that has two levels of frequency. The generated sound is a sine waveform that switches between 1 kHz and 2 kHz with a sampling rate of 44.1 kHz. We generated the sound audio file from [59] and the generated sound audio file shown in Figure 3.

### 5.2. Mobile Phones

We used twenty-five mobile phones to capture the sound (noise) level. Each mobile phone is an iPhone 14 Pro running iOS 17.5.1 and has an installed purchased sound level meter (Decibel: dB Sound Level Meter—Premium Mode version 9.3.1). All records were considered in the experiment, as all mobile phones were identical and no issues were reported.

### 5.3. Test Location

**Figure 4:**
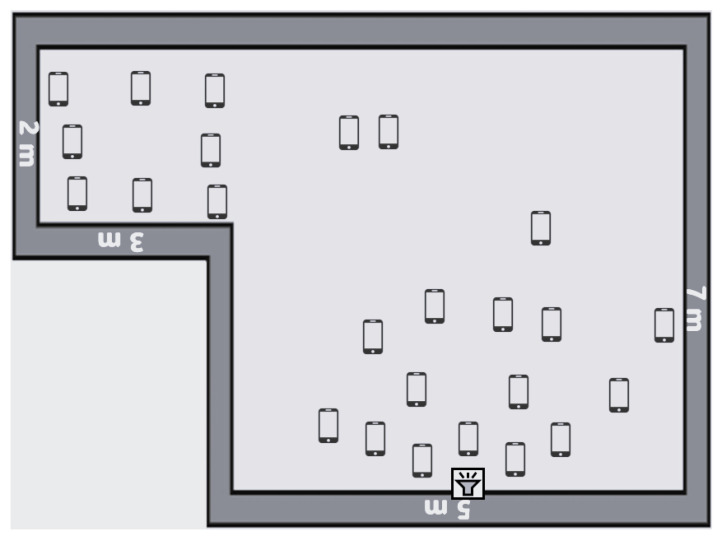
Room layout.

We conducted the experiment in a 7 m × 5 m room attached to a 3 m × 2 m corridor. We placed the sound generator in the middle of one wall and distributed the mobile phones across the room and corridor, as shown in Figure 4. We recorded the coordinates of the sound source location (in the middle of the 5 m wall at the bottom of Figure 4) and normalized them to be the center or typically the coordinates x = 0, y = 0. Afterward, we recorded the coordinates of each mobile device and normalized the coordinates according to the sound source location. The coordinates of each mobile device and the position of the sound generator are shown in Table A1 in Appendix A.

### 5.4. Clustering Algorithm

**Figure 5:**
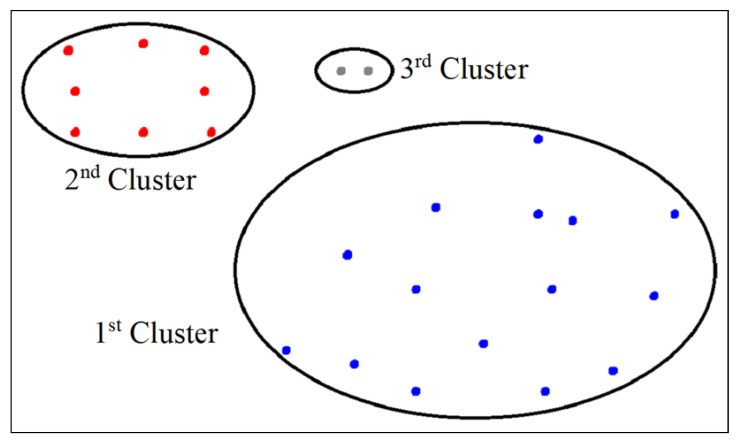
The spatial clusters generated via DBSCAN.

We used the DBSCAN algorithm as a clustering tool for both clustering phases, namely, spatial clustering and noise level clustering. On the one hand, for spatial clustering, the DBSCAN settings were ϵ=2 m and MinPoints=2, that is, we needed a minimum of 2 mobile phones with distances of less than 2 m to form a spatial cluster. On the basis of the mentioned DBSCAN settings, the formed spatial clusters are shown in Figure 5. The first subset is located very close to the sound source and contains 15 mobile phones. The second subset is located in the corridor area and contains 8 mobile phones. The third subset is located at the top–middle of the room and contains 2 mobile phones. On the other hand, for noise level clustering, the DBSCAN settings were ϵ = 3 dB and MinPoints=2, that is, we needed a minimum of 2 mobile phones with a difference of less than 3 dB in noise level reading to form a noise level cluster. These settings could be refined and tuned experimentally for best practice.

## 6. Results

**Figure 6:**
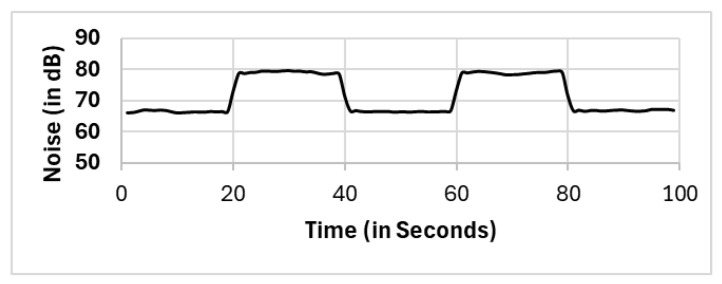
Full population (FP).

**Figure 7:**
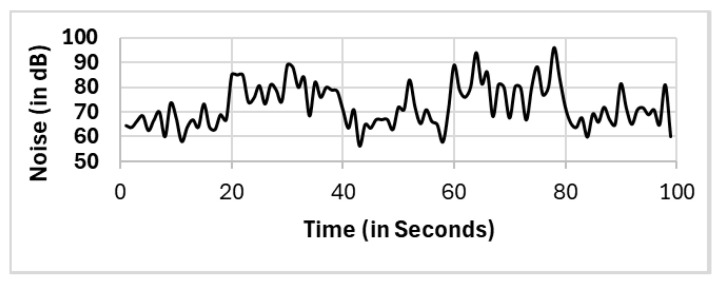
Randomly selected single mobile (RS).

**Figure 8:**
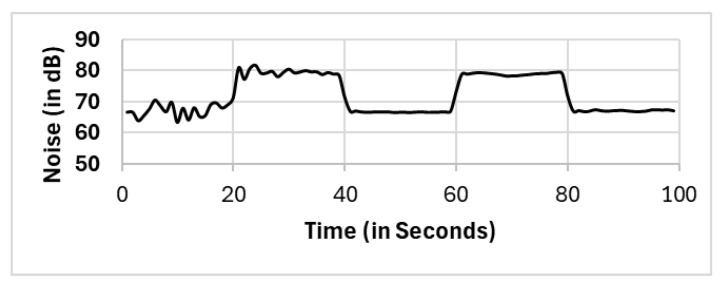
Subset selection (SS).

**Figure 9:**
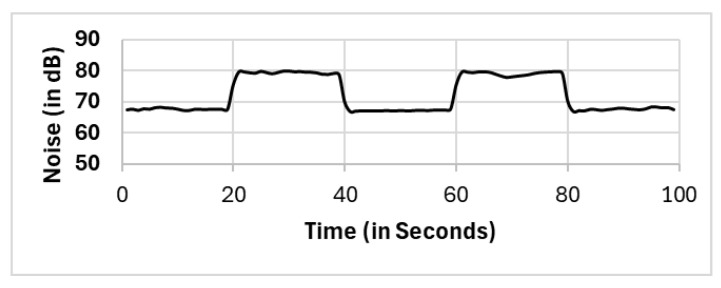
C_spatial_(c1).

**Figure 10:**
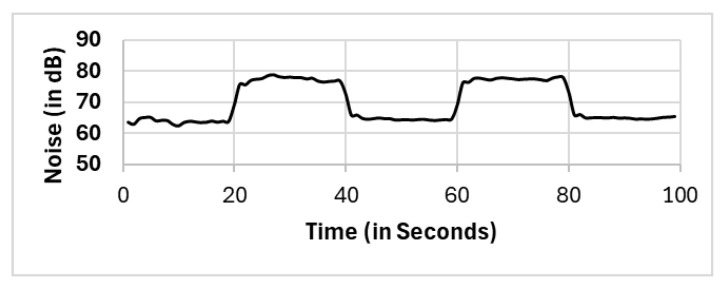
C_spatial_(c2).

**Figure 11:**
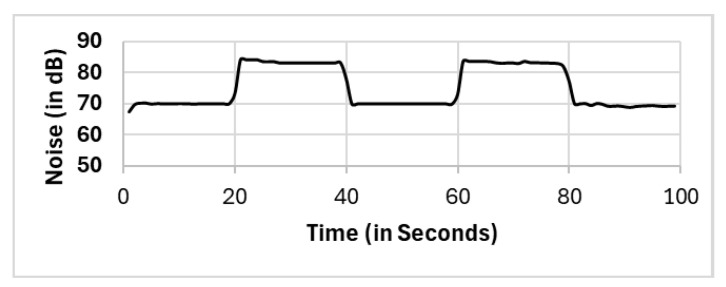
C_spatial_(c3).

**Figure 12:**
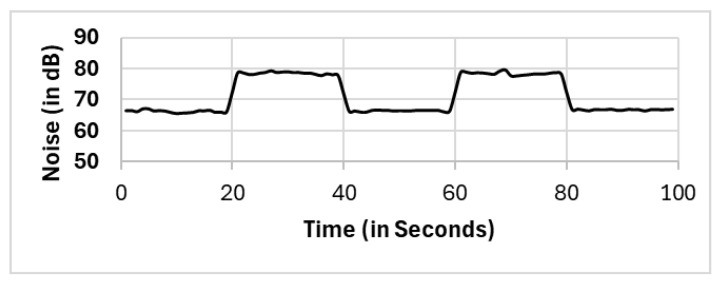
C_Noise_.

**Figure 13:**
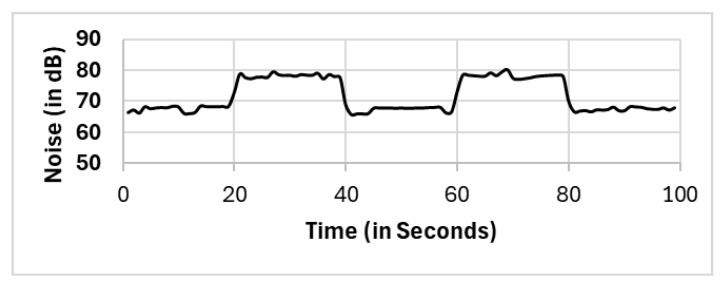
C_spatial_(c1)⇒C_Noise_.

**Figure 14:**
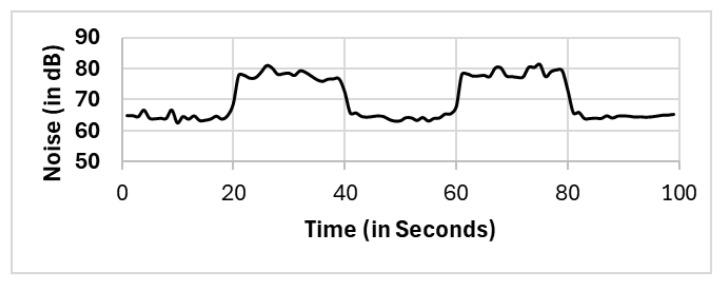
C_spatial_(c2)⇒C_Noise_.

In this section, we present the reported results for all the selection methods, including our proposed approach, and the variations among them. The methods include full population (FP), randomly selected single mobile (RS), subset selection (SS), noise clustered (C_Noise_), spatially clustered (C_Spatial_), and our proposed two clustering phases (C_Spatial_⇒C_Noise_). The reported readings according to each selection method FP, RS, SS, C_Spatial_ (cluster 1), C_Spatial_ (cluster 2), C_Spatial_ (cluster 3), C_Noise_, C_Spatial_⇒C_Noise_ (cluster 1), and C_Spatial_⇒C_Noise_ (cluster 2) are shown in Figure 6, Figure 7, Figure 8, Figure 9, Figure 10, Figure 11, Figure 12, Figure 13 and Figure 14, respectively.

In a mathematical representation, we demonstrate the statistical process of our proposed two clustering phases (C_Spatial_⇒C_Noise_), for example, at time (t=21). According to Equation (18), the set of mobile phones to be recruited is shown below Equation (26):

(26) $$c_{X,Y}^{spatial,noise}\left(t_{21}\right)=\left[\begin{array}{c} \left\{m_{2},m_{4},m_{5},m_{6},m_{7},m_{9},m_{10},m_{11},m_{12},m_{13},m_{14},m_{15}\right\}\\ \left\{m_{16},m_{18},m_{19},m_{20},m_{21},m_{22},m_{23}\right\}\\ \left\{m_{24},m_{25}\right\} \end{array}\right]$$

According to Equation (21), the set of outliers is shown below Equation (27):

(27) $$Outliers\left(t_{21}\right)=\left\{P^{spatial},Q^{noise}\right\}=\left[\begin{array}{c} P^{spatial}\\ Q_{1}^{noise}\\ Q_{2}^{noise}\\ Q_{3}^{noise} \end{array}\right]=\left[\begin{array}{c} \phi ,\\ \{m_{1},m_{3},m_{8}\},\\ \{m_{17}\},\\ \phi \end{array}\right]$$

All methods, except the randomly selected single-mobile (RS) method, yield fair readings following the trends of the sound source (as shown in Figure 3). However, there are variations in the reported noise levels among methods; therefore, we compared these variations with respect to the full population method (FP). We captured the variations as high/low/average differences between the full population (FP) method and the other methods.

**Figure 15:**
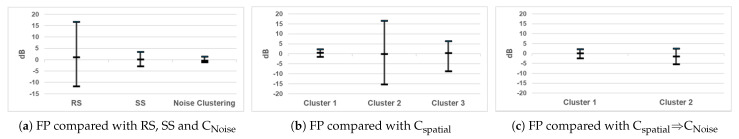
Full population (FP) compared with the other selection methods: (**a**) with random selection (RS), subset selection (SS) and noise clustered (C_Noise_), (**b**) with spatial clusters (C_spatial_), and (**c**) with the proposed two−phase clustering method (Cspatial⇒C_Noise_).

In Figure 15a, we plotted the differences (in dB) between the readings from the full population (FP) method and the readings from the randomly selected single-mobile (RS), subset selection (SS), and noise clustering methods. Each vertical line represents three numbers: the top line represents the maximum difference reported, the bottom line represents the minimum difference reported, and the middle line represents the average difference reported.

We noted that there were large differences between the full population (FP) method and the randomly selected single-mobile method. We observed variations that reached 16.7 dB higher and 11.7 dB lower than the reported readings in full population methods. This variation is narrower than that of the other two methods, namely, the subset selection (SS) method and the noise clustering (C_Noise_) method. The variations were within 5 dB.

We compare the full population (FP) method with the spatial clustering (C_Spatial_) method. Practically, we compare the full population (FP) with each formed cluster, namely, cluster 1 (15 mobile phones), cluster 2 (8 mobile phones) and cluster 3 (2 mobile phones). On the one hand, we observed that the variation was small between the full population method and cluster 1, and the variation was less than 5 dBs. On the other hand, substantial variations between the full population method and the other two clusters (clusters 2 and 3) were observed. The variations between the full population (FP) method and cluster 2 and cluster 3 were approximately 15 dB and 10 dBs, respectively.

Finally, we compared the full population (FP) method with the proposed method (C_Spatial_⇒C_Noise_). We compare the full population (FP) method with cluster 1 and cluster 2, which are formed by the first phase of the clusters. Cluster 3 contains only two mobile phones, and there are no outliers according to the second clustering phase. That is to say, the set of mobile phones is similar to the spatial clustering (C_Spatial_ method. The variation between the FP method and cluster 1 was fair and occurred within 5 dBs, whereas cluster 2 presented greater variation above 5 dBs. We noticed that cluster 2 shows readings (on average) lower than those of the full population (FP) methods.

## 7. Discussion

MCS platform data collection can be comprehensive or selective, employing techniques such as clustering to focus on participant segments. In our experiment, we applied different methods to calculate the noise level. We used as a reference the well-known deployed method, which is the full population (FP), and existing methods, such as randomly selected single-mobile (RS), subset selection (SS), spatially clustered (C_Spatial_), noisy clustered (C_Noise_), and our proposed method, which has two clustering phases (C_Spatial_⇒C_Noise_). Each of the methods has advantages and disadvantages; however, we are interested in the variation between the methods in reporting the noise level. In the following, we highlight the main observations, with reference to the full population (FP) method.

### 7.1. T Test Comparisons Among Selection Methods

In this work, we examined five major selection methods, namely, the FP, RS, SS, C_Spatial_ and C_Noise_ methods, and our proposed C_Spatial_⇒C_Noise_ method. To reach a thorough and careful comparison among these methods, we need to declare a precise null hypothesis that could/could not initially support the needs of our proposed method. Therefore, we suggest the following null hypothesis ($H_{0}$).

**Hypothesis 1**  (Null Hypothesis—$H_{0}$). *When noise level readings are collected via mobile phones that are diffusionally scattered in an (indoor) area, even when a clustering algorithm is applied, **NOT** results in a vital accuracy degradation of the reported noise level.*

The null Hypothesis 1 could be evaluated via a paired *t* test among selection methods with a two-tailed test. The degree of freedom is 98, the significance level is 0.05 (95%), and the T value (or the critical value) is ±1.9845. We used the SciPy (https://scipy.org/ accessed on 10 January 2025) library in Python, version 3.13.2. (Stichting Mathematisch Centrum, Amsterdam, the Netherlands). to perform the *t* test among the selection methods, and the reported outcomes are shown in Table 2. In Table 2, there are three columns, namely, the methods in comparison (first column), statistic test value (second column), and *p* value (third column). The statistic test value is used to identify statistically if there is a notable variation between the means of the two methods in the comparison. If the absolute value of the statistical test value is high, then there is a potential difference between the two methods; specifically, the means of the two methods differ significantly. The *p* value (*p*) is a probability value that indicates how much the results could be due to random chance. In our comparison, we set a threshold of 0.05 as the significance level (α) at which our Hypothesis 1 can be rejected, that is, p≤α. As a result, if the *p* value (*p*) is less than or equal to the significance level (α), then we can reject Hypothesis 1 (null hypothesis), and vice versa.

From the outcomes of the *t* test, particularly the *p* values, which are below the significance level, we can infer that neither the traditional full population (FP), randomly selected single mobile (RS), and subset selection (SS) nor single-phase clustering (C_Spatial_ and C_Noise_) were close comparisons. The *p* values were almost zero for all comparisons among the selection methods, which resulted in rejection of the null hypothesis (Hypothesis 1). As a result, we are sure that there is a vital accuracy degradation of the reported noise level among the selection methods on the MCS platform. This finding indicates that careful selection of mobile phones as noise level-reporting devices in MCS platforms is critical. Applying standard spatial clustering would not be enough to capture the variations, as we can see in the table that our proposed method (C_Spatial_⇒C_Noise_) shows variations less than the significance level 0.05 compared with spatial clustering (C_Spatial_).

### 7.2. Cluster Location

The location of mobile phones and the distance from the noise or sound source have a significant impact, which implies the need for clustering algorithms on the basis of spatial location. In our experiment, after we apply the DBSCAN algorithm to the spatial domain, we have three clusters, namely, clusters 1, 2 and 3. The variations between clusters 2 and 3 (Figure 10 and Figure 11, respectively) and the reference method (FP) (Figure 6) were very clear, as reported in Figure 15b. This variation (gap) results from the distance variation impacting the sound intensity, as given by Equation (1). This variation in the readings among clusters implies the need for spatial clustering prior to reporting the noise level in MCS platforms. In the proposed two clustering phases, spatial clustering is implemented in the first clustering phase, and the variations are limited, as shown in Figure 15c.

### 7.3. Outliers

**Figure 16:**
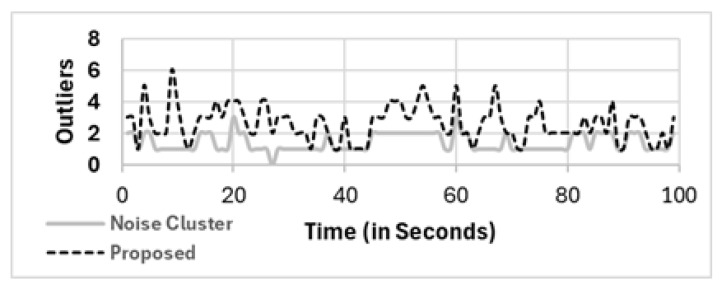
Outliers detected and eliminated by noise cluster (C_Noise_) denoted as **noise cluster** on the gray line and our proposed two clustering phases (C_Spatial_⇒C_Noise_) denoted as **Proposed** on the dashed line.

Although all the mobile phones in our study were able to read the sound pressure level (SPL), depending on the location of some mobile phones, the readings of the sound level (or noise level) for some mobile phones were far from the others. The readings from these devices are considered outliers, and they are not considered in the analysis. In the full population (FP) method as well as other methods, i.e., RS, SS, and C_Spatial_, these outliers were considered in the noise level calculation. Only C_Noise_ and our proposed method’s two clustering phases were able to eliminate them, as shown in Equation (19) for our proposed method. Figure 16 shows the number of eliminated mobile phones as outliers in the noise cluster (C_Noise_) and our proposed two−phase clustering method (C_Spatial_⇒C_Noise_, denoted as proposed in the figure). Our proposed selection method is more efficient at eliminating outliers than other clustering methods. Compared with noise clustering, 4% to 12% more outliers are detected and eliminated (C_Noise_), which is much greater than the 20% of the population of mobile phones in the area of interest.

**Figure 17:**
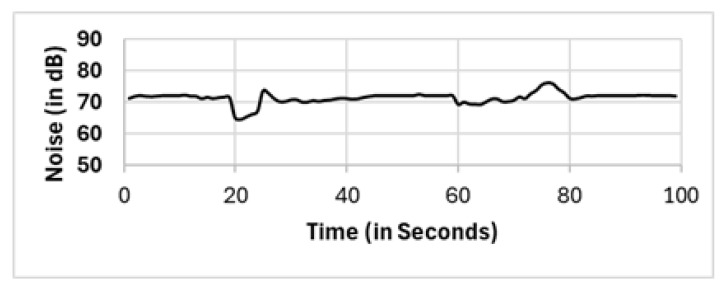
Readings from mobile phone 8, which is located at x: −2.88 and y: 0.84.

In most existing works, these outliers, which are located in critical positions, are vital outliers, as they are not spatial outliers but feature-based (noise level) outliers. These noise level outliers have a considerable impact on the calculations. We recall that the nature of DBSCAN is capturing the density in the spatial domain. Therefore, the formation of clusters via the DBSCAN algorithm will only generate clusters on the basis of the density of the location. Existing works [60,61] use multifeature DBSCAN, which involves normalization of observations prior to applying the DBSCAN algorithm. However, our proposed two clustering phases detect and eliminate outliers in spatial clustering and then feature-based “noise level” clustering. In Figure 17, the noise level reported by one of the “noise level” outliers, which is fairly close to the sound source (mobile number 8 with coordinates x: −2.88 and y: 0.84 from the source; refer to Table A1), does not show normal noise trending as the sound generated in Figure 3. This finding supports our proposed method, which requires two clustering phases to eliminate not only the spatial outliers but also the featured “noise level” outliers.

### 7.4. Applications

According to the noise mapping process described in [53], the collected data must be subjected to noise calculation and validation, followed by the construction of noise mapping based on noise interpolation. Afterward, the process continues to estimate the exposure of the population to noise, and consequently, noise actions may be needed in public health policies. Our proposed selection method, i.e., the two−phase clustering method, is an essential step for environmental noise mapping and noise control. The outcome of clustered mobile phones with eliminated outliers in a focused area will support the noise mapping process. Through our proposed selection method, the processing steps could be shortened as the noise calculation and validation are completed with greater accuracy.

Furthermore, the formed spatial clusters with omitted outliers are potentially helpful as metadata in noise mapping. Studying the metadata of the formed clusters, such as the centroid of the cluster, the cluster size, and the cluster coverage, would lead to identifying the noise source in the spatial domain. Moreover, these metadata of the formed clusters support the assessment of noise control and environmental noise policy enforcements in buildings, industries, and urban areas.

## 8. Conclusions

MCS platforms for noise monitoring are promising technologies for noise pollution control in smart buildings and smart cities. Apparently, there is a risk of false reporting of noise pollution if the statistical analysis and mobile phone selection methodology are not conducted carefully. We hypothesize that the selection methodology involved in recruiting mobile phones for collecting noise level readings on the MCS platform will not effectively report the accuracy of noise pollution.

In this paper, we explore the pitfalls that could occur on an MCS platform for noise monitoring, which is the selection methodology among participant mobile phones. We discuss five (5) selection methods as well as our proposed two clustering phases for noise level monitoring. We evaluated the five selection methods and our proposed method through a statistical evaluation, namely, a *t* test. The *t* test shows that all existing selection methods, namely, the full population, randomly selected single-mobile, subset selection, noise clustering and spatial clustering selection methods, do not report information that is the same or close to each other. This is rooted in the nature of acoustics, as well as the outlier mobile phone readings affecting the reported noise level.

Moreover, we evaluate our proposed method with the noise clustering method in terms of its performance in detecting and eliminating outliers from the calculation. Our proposed method shows better performance in detecting and eliminating outlier mobile phones, with 4% to 12% improvement over the noise clustering method.

In the future, we will explore more clustering algorithms to provide rigorous insights and in-depth analysis. Although machine learning clustering algorithms have been examined as suggested in several works, i.e., mobile crowd sensing low-energy clustering (MCLEC) [62], with density-based spatial clustering of applications with noise (DBSCAN) [14], we argue that extended versions of MCLEC, DBSCAN, hierarchical agglomerative clustering [63,64], fuzzy clustering [65], and k-means clustering could have potential in supporting noise monitoring based on crowd-sourced mobile phones. In particular, hierarchical agglomerative clustering has a greater possibility for forming clusters with tunable thresholds.

## Figures and Tables

**Table 1:** A summary of related studies on MCS-based noise-monitoring systems.

Reference	Research Objective and Scenario	Density	Centroid of Density	Population (Full or Clustered)	Outliers Included in the Analysis
[46,47]	Emerging SPL meters with MCS platform and spatial data for noise mapping	High	Not considered	Full Population	Included
[48]	Evaluating the uncertainty in MCS-based reported noise	High	Considered	Full Population	Partially Not Included
[11,14]	Developing a survey-based tool for noise monitoring	Individual	Not considered	Full Population	Included
[49]	MCS-based platform for noise monitoring on campuses	High	Not considered	Full Population	Included
[50]	Developing an iOS tool for noise thermal monitoring	Normal	Not considered	Full Population	Included
[51]	Calibrating method for mobiles on MCS platform	High	Considered	Full Population	Included
[52]	Indoor localizing mobile using sound	Individual	Not considered	Single	N/A
[13]	Noise monitoring at city scale using merged GIS and MCS platform	High	Considered	Clustered	Included

**Table 2:** *t*-test comparison among selection methods on MCS platform. The degree of freedom (DoF) is 98, the significance level 0.05, and the T value (critical value) is ±1.9845.

Methods in Comparison	Statistic Test	*p* Value
FP vs. RS	−7.331550833	0.00000000006570
FP vs. SS	−7.735057609	0.00000000000928
FP vs. C_spatial_($c_{1}$)	−7.345379876	0.00000000006146
FP vs. C_spatial_($c_{2}$)	−7.460360552	0.00000000003527
FP vs. C_spatial_($c_{3}$)	−6.77181776	0.00000000094629
FP vs. C_Noise_	−8.461310722	0.00000000000026
FP vs. C_spatial_(c1) ⇒ C_Noise_	−7.201602315	0.00000000012269
FP vs. C_spatial_(c2) ⇒ C_Noise_	−7.76173871	0.00000000000815
C_spatial_(c1) vs. C_Noise_	−25.11997476	0.00000000000000
C_spatial_(c2) vs. C_Noise_	−13.28195365	0.00000000000000
C_spatial_(c1) vs. C_spatial_(c1) ⇒ C_Noise_	2.302621708	0.02341503193879
C_spatial_(c2) vs. C_spatial_(c2) ⇒ C_Noise_	−3.355382514	0.00112858122471
C_Noise_ vs. C_spatial_(c1) ⇒ C_Noise_	15.86083916	0.00000000000000
C_Noise_ vs. C_spatial_(c2) ⇒ C_Noise_	11.81506333	0.00000000000000

## Data Availability

The author declare that the data supporting the findings of this study are available at https://doi.org/10.3886/E217001V2.

## Abbreviations

The following abbreviations are used in this manuscript:

MCS	Mobile crowd sensing
FP	Full population
RS	Randomly selected single mobile
SS	Subset selection
C_Spatial_	Spatial clustered
C_Noise_	Noise level clustered
C_Spatial_⇒C_Noise_	proposed two clustering phases

## Appendix A. Coordinates of Mobile Phones

The coordinates of the 25 mobile phones used in the experiment are listed in Table A1. The readings are available online at [58].

**Table A1:** Coordinates of mobile phones.

Mobile No.	X	Y
1	0.97	0.23
2	0	0.97
3	−0.98	0.2
4	1.96	0.541
5	1.05	1.7
6	−0.91	1.78
7	−1.9	0.64
8	−2.88	0.84
9	−1.96	2.27
10	−0.64	2.93
11	1.31	2.7
12	2.52	1.62
13	0.89	2.86
14	2.87	2.84
15	0.88	3.9
16	−6	4
17	−6.05	5.21
18	−4.97	4.04
19	−4.97	5.3
20	−4	4
21	−4.07	5.21
22	−4.02	4.605
23	−5.98	4.69
24	−2.05	4.96
25	−1.69	4.91
